# Evaluation of the Accuracy of Asprosin as a Biomarker in Breast Cancer

**DOI:** 10.3390/biomedicines14030498

**Published:** 2026-02-25

**Authors:** Ozlem Unal, Servet Kocaoz, Kamil Osma, Funda Eren

**Affiliations:** 1 Department of Radiology, Ankara Yıldırım Beyazıt University, Ankara 06300, Türkiye; 2 Department of General Surgery, Ankara Bilkent City Hospital, Ankara 06530, Türkiye; servet.kocaoz@gmail.com; 3 Department of General Surgery, Ankara Polatlı State Hospital, Ankara 06900, Türkiye; kamilosma55@gmail.com; 4 Department of Biochemistry, Istanbul Basaksehir Cam ve Sakura City Hospital, Istanbul 34480, Türkiye; fundakarakoyunlu@gmail.com

**Keywords:** asprosin, breast cancer, biomarker, ELISA, diagnosis, tumor stage, hormone

## Abstract

**Background:** Asprosin is a fasting-induced adipokine involved in metabolic regulation and has recently been implicated in cancer biology. However, data regarding its diagnostic accuracy and clinicopathological associations in breast cancer remain limited. **Methods:** Serum asprosin levels were measured in patients with breast cancer and healthy controls using ELISA. Associations between serum asprosin levels and clinicopathological parameters were analyzed, and accuracy performance was evaluated using receiver operating characteristic (ROC) curve analysis. **Results:** Serum asprosin levels were significantly higher in the breast cancer group compared with controls (14.3 ng/mL vs. 11.0 ng/mL; *p* < 0.001). ROC curve analysis identified an optimal cutoff value of 12.2 ng/mL based on the Youden index, yielding a sensitivity of 66.4% and a specificity of 65.5%, indicating statistically significant yet moderate accuracy performance. A statistically significant difference in serum asprosin levels was observed according to HER2 status, smaller tumor size, and early-stage disease (*p* < 0.05). Serum asprosin levels tended to be higher in patients without axillary lymph node metastasis; however, this difference did not reach statistical significance (*p* = 0.050). No significant associations were observed with estrogen receptor positivity or the Ki-67 proliferation index. Serum asprosin levels were also correlated with body mass index and breast cancer presence (Spearman’s rho, *p* < 0.01). **Conclusions:** Serum asprosin levels are elevated in patients with breast cancer and demonstrate moderate accuracy performance. Increased levels observed in early-stage disease suggest that asprosin may reflect early tumor biological characteristics rather than overall tumor burden. Further large-scale and longitudinal studies are required to validate its clinical utility.

## 1. Introduction

Breast cancer is the most frequently diagnosed malignant tumor among women worldwide and continues to represent a major public health burden in both developed and developing countries. According to data from the World Health Organization, one in eight women will develop breast cancer during her lifetime. Early diagnosis significantly improves treatment outcomes and markedly reduces disease-related mortality. Therefore, the identification and validation of new and reliable biomarkers for early diagnosis and prognosis are of critical importance for improving clinical management and patient survival [1].

Since its identification by Romere and colleagues in 2016, asprosin has increasingly attracted scientific interest, particularly in the fields of metabolism and cancer biology [2,3]. Asprosin is an adipokine that primarily plays a role in glucose homeostasis and energy metabolism, and alterations in its circulating levels have been reported in various endocrine and metabolic disorders, including obesity, insulin resistance, diabetes mellitus, and polycystic ovary syndrome. Beyond metabolic regulation, a growing body of evidence suggests that asprosin may also play a role in tumor development. Dysregulated asprosin expression has been observed in various solid tumors, including colon, pancreatic, ovarian, and breast cancer. In studies focusing specifically on breast cancer, asprosin expression has been shown to be higher in tumor tissues compared with normal breast tissue, indicating a potential role for asprosin in tumor development and progression [4].

Despite these emerging findings, data regarding the diagnostic accuracy of asprosin in breast cancer remain limited. In particular, studies evaluating the relationship between serum asprosin levels and the clinicopathological characteristics of breast cancer are scarce. Therefore, further investigations are needed to clarify the potential role of asprosin as a diagnostic biomarker and to better understand its association with tumor biology.

The primary aim of this study was to evaluate the relationship between serum asprosin levels and menopausal status, body mass index, histopathological tumor characteristics, disease stage, and molecular subtypes in patients diagnosed with breast cancer. In addition, the study aimed to determine the accuracy performance of serum asprosin and to investigate its potential applicability as a clinical adjunct biomarker in breast cancer.

## 2. Materials and Methods

### 2.1. Ethical Approval and Study Design

This prospective case–control study was conducted after obtaining approval from the Ankara Bilkent City Hospital TABED Ethics Committee No. 1 (approval date: 13 November 2024; approval number: TABED 2-24-526). All procedures carried out within the scope of the study were performed in accordance with institutional research ethics standards and the principles of the Declaration of Helsinki.

### 2.2. Study Population

Patients who presented to the General Surgery Clinic of Ankara Bilkent City Hospital between 1 December 2024 and 1 April 2025 were evaluated for eligibility. Following radiological assessment performed at the Breast Radiology Imaging Center, a total of 110 patients who were diagnosed with breast cancer and scheduled for surgical treatment were included in the patient group.

The control group consisted of 110 women who presented to the Breast Endocrine Surgery outpatient clinic for routine breast evaluation and whose imaging findings were classified as BI-RADS 1 or BI-RADS 2.

Prior to inclusion in the study, all participants were informed about the study protocol, and written informed consent was obtained. Exclusion criteria included age under 18 years, male sex, radiological or pathological examinations performed at external centers, refusal to participate in the study, receipt of neoadjuvant chemotherapy, and presence of systemic inflammatory disease. Accordingly, two patients with rheumatoid arthritis, twelve patients diagnosed with diabetes mellitus, and one patient with a concomitant malignancy other than breast cancer were excluded from the control group. In addition, three breast cancer patients whose biopsy procedures were performed at external centers were excluded from the patient group.

### 2.3. Data Collection

For patients in the breast cancer group, data including age, body mass index, menopausal status, type of surgical procedure performed, histological tumor subtype, tumor grade, tumor size, axillary lymph node metastasis status, presence of ductal carcinoma in situ, disease stage, and radiological imaging findings were obtained from pathology reports and electronic medical records.

For participants in the control group, age, body mass index, menopausal status, and radiological imaging results were recorded.

#### Assessment of ER, PR, HER2, and Ki-67 Status

Estrogen receptor (ER) and progesterone receptor (PR) status were assessed by immunohistochemical analysis, and nuclear staining in ≥1% of tumor cells was accepted as positive, in accordance with current clinical guidelines. Human epidermal growth factor receptor 2 (HER2) status was evaluated using immunohistochemical scoring; cases with a score of 3+ were considered HER2 positive, while cases with a score of 2+ were further evaluated by fluorescence in situ hybridization (FISH), and final HER2 status was determined based on FISH results. The Ki-67 proliferation index was defined as the percentage of positively stained tumor cell nuclei and was classified as low (<14%) or high (≥14%) proliferative activity using a cutoff value of 14%.

### 2.4. Measurement of Serum Asprosin Levels

Venous blood samples were collected from both breast cancer patients and healthy controls into gel-containing serum tubes. Samples were allowed to clot at room temperature for approximately 20 min, followed by centrifugation at 1300× *g* for 10 min. The resulting serum was carefully aliquoted and stored at −80 °C until biochemical analysis.

To minimize preanalytical variability, all blood samples were obtained after confirming fasting status. According to the fasting standardization applied, the effects of short-term hormonal fluctuations related to acute nutritional status were minimized. In addition, hemolyzed, lipemic, or icteric specimens were excluded. All serum samples were stored at −80 °C for a maximum of three months and thawed only once prior to ELISA analysis.

After all samples were collected, serum asprosin measurements were performed in a single analytical batch by a clinical biochemistry specialist in our hospital’s central biochemistry laboratory, ensuring analytical consistency.

Serum asprosin concentrations were measured using a quantitative double-antibody sandwich ELISA with a commercially available Human Asprosin ELISA Kit (BT Lab, Bioassay Technology Laboratory, Jiaxing, China; lot no: 202502008). The analytical characteristics provided by the manufacturer were as follows:

- Analytical range: 0.5–100 ng/mL;
- Sensitivity: 0.23 ng/mL;
- Intra-assay CV: <8%;
- Inter-assay CV: <10%.

### 2.5. Statistical Analysis

Statistical analyses were performed using the Statistical Package for the Social Sciences (SPSS) software, version 25.0 (IBM Corp., Armonk, NY, USA). Categorical variables were summarized using descriptive statistics and reported as frequencies and percentages. Continuous variables were expressed as median [interquartile range] due to violation of the normality assumption; minimum and maximum values were reported only where clinically relevant. The normality of data distribution was assessed using the Kolmogorov–Smirnov test. Normality assumption for age, body mass index (BMI), and serum asprosin levels was violated. Height and weight measurements were obtained for BMI calculation; however, BMI was selected as the primary anthropometric variable for comparative statistical reporting.

Age categorization was based on the median value of the study cohort to ensure balanced subgroup sizes for nonparametric comparison. Comparisons between categorical variables were conducted using the chi-square test. For non-normally distributed continuous variables, between-group comparisons were performed using the Mann–Whitney U test for two groups and the Kruskal–Wallis test for comparisons among more than two groups. When the Kruskal–Wallis test indicated a statistically significant overall difference, post hoc pairwise comparisons were conducted. Each pairwise comparison tested the null hypothesis that the distributions of the two groups were the same. All *p*-values were two-sided. To control for multiple testing, the Bonferroni correction was applied and Bonferroni-adjusted *p*-values were reported. The statistical significance level was set at 0.05.

Receiver operating characteristic (ROC) curve analysis was performed to evaluate the accuracy performance of serum asprosin levels for discriminating breast cancer patients from healthy controls. The optimal cut-off value was determined using the Youden index, and corresponding sensitivity, specificity, positive predictive value, and negative predictive value were calculated.

## 3. Results

### 3.1. Clinicopathological Characteristics

Among patients diagnosed with breast cancer, the predominant histological subtype was ductal carcinoma, NST, which was identified in 93.6% of patients (*n* = 103). Invasive lobular carcinoma was detected in 6.4% of patients (*n* = 7) (Table 1). The presence of concomitant ductal carcinoma in situ (DCIS) was identified in 21.8% of patients (*n* = 24).

With respect to tumor grade, 14.3% of tumors (*n* = 10) were classified as grade 1, 70.0% (*n* = 49) as grade 2, and 15.7% (*n* = 11) as grade 3.

Estrogen receptor (ER) positivity was detected in 85.5% of patients (*n* = 94), progesterone receptor (PR) positivity in 80.0% (*n* = 88), and human epidermal growth factor receptor 2 (HER2) positivity in 30.0% (*n* = 33). The proportion of patients with a Ki-67 proliferation index below 14% was 34.5% (*n* = 38), whereas the proportion with a Ki-67 proliferation index of 14% or higher was 65.5% (*n* = 72).

Axillary lymph node metastasis was not detected in 41.8% of patients (*n* = 46), whereas metastasis was present in 58.2% (*n* = 64). Distant organ metastasis (liver or bone) was detected in only 3.6% of patients (*n* = 4).

When surgical treatments were evaluated, 60.0% of patients (*n* = 66) underwent breast-conserving surgery with sentinel lymph node biopsy (BCS + SLNB), 0.9% (*n* = 1) underwent breast-conserving surgery with axillary lymph node dissection (BCS + ALND), 20.9% (*n* = 23) underwent mastectomy with sentinel lymph node biopsy, and 18.2% (*n* = 20) underwent modified radical mastectomy.

Early-stage disease (Stage I–II) was present in 73.6% of patients (*n* = 81) (Table 1).

### 3.2. Demographic Characteristics

The median age of patients was 51 years [IQR: 45–62], whereas the median age of the control group was 49 years [IQR: 45–53]. The median body mass index (BMI) was 25.8 kg/m^2^ [IQR: 23.7–30.0] in patients with breast cancer and 25.75 kg/m^2^ [IQR: 23.7–29.2] in the control group. BMI was used as the primary anthropometric parameter in comparative analyses.

### 3.3. Serum Asprosin Levels

The median serum asprosin level was 14.3 ng/mL [IQR: 11.44–22.6] in the breast cancer group. In the control group, the median serum asprosin level was 11.0 ng/mL [IQR: 9.16–13.56].

When demographic variables were compared, the control group was significantly younger than the breast cancer group (*p* = 0.014) (Table 2). No statistically significant difference was observed between groups in terms of body mass index (BMI) (*p* = 0.909). BMI was used as the primary anthropometric indicator in the comparative analysis.

**Figure 1:**
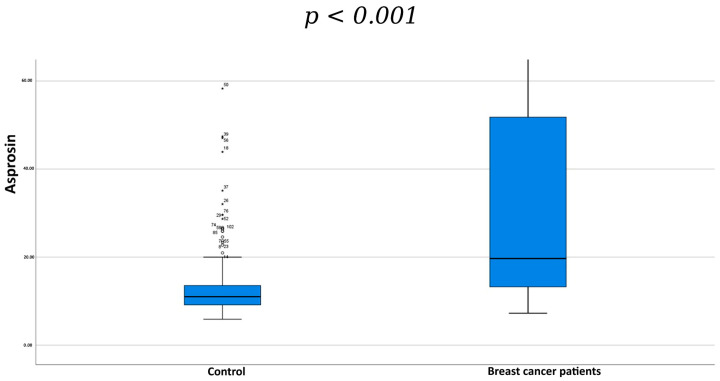
Comparison of serum asprosin levels between breast cancer patients and the control group. Data are shown as median and interquartile range (IQR). Statistical analysis was performed using the Mann–Whitney U test. A statistically significant difference was observed between the two groups (*p* < 0.001). The asterisk (*) and dot indicate statistically significant pairwise differences between the specified groups based on post hoc analysis.

In contrast, serum asprosin levels were significantly higher in the breast cancer group compared with controls (*p* < 0.001) (Figure 1). Comparisons based on menopausal status showed no statistically significant difference between groups (*p* = 0.058) (Table 2).

### 3.4. Accuracy Performance of Serum Asprosin

**Figure 2:**
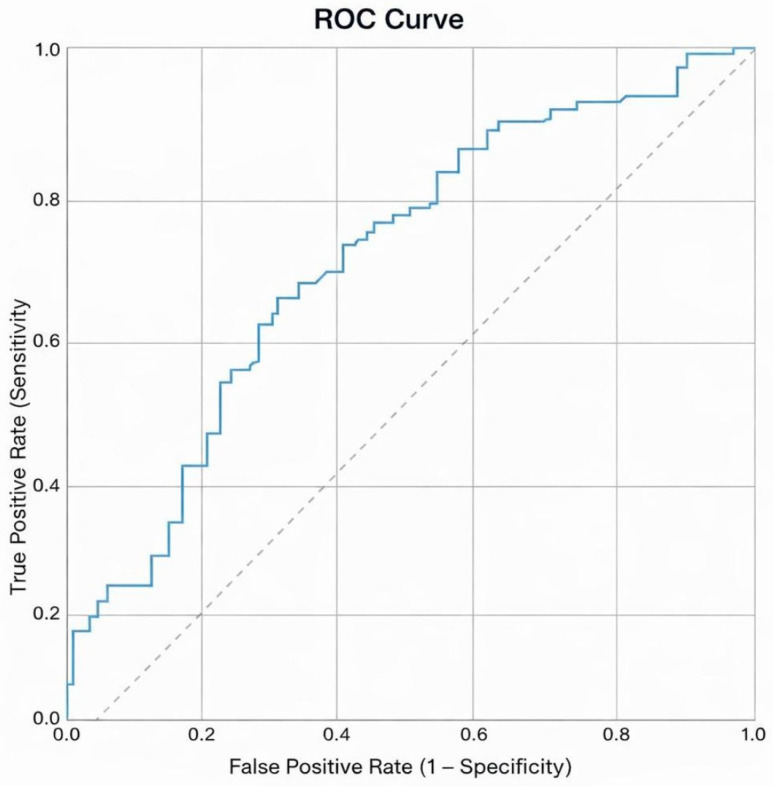
Receiver operating characteristic (ROC) curve analysis of serum asprosin levels for discriminating breast cancer patients from the control group. The optimal cut-off value was determined.

ROC curve analysis was performed to evaluate the accuracy performance of serum asprosin levels for breast cancer (Figure 2). The optimal cutoff value was identified as 12.2 ng/mL. At this cutoff, serum asprosin levels were significantly associated with breast cancer presence (*p* < 0.001) (Table 3).

At this threshold, sensitivity was 66.4% and specificity was 65.5%. The positive predictive value was 65.8%, and the negative predictive value was 66.1%. These findings indicate statistically significant but moderate accuracy performance. Accordingly, serum asprosin should not be interpreted as a standalone diagnostic test but may represent a potential complementary biomarker.

### 3.5. Relationship Between Serum Asprosin Levels and Tumor Characteristics

Serum asprosin levels were significantly higher in patients with progesterone receptor negativity, HER2 negativity (Table 4), smaller tumor diameter, and early-stage disease (Table 5) (*p* = 0.034; *p* = 0.033; *p* < 0.001; *p* = 0.02, respectively). In patients without axillary lymph node metastasis, serum asprosin levels were numerically higher; however, this difference was borderline and did not reach statistical significance (*p* = 0.050).

Although a numerical difference in serum asprosin levels was observed according to estrogen receptor (ER) status, this difference did not remain statistically significant after correction for multiple testing and was therefore not considered statistically significant (*p* = 0.083).

**Figure 3:**
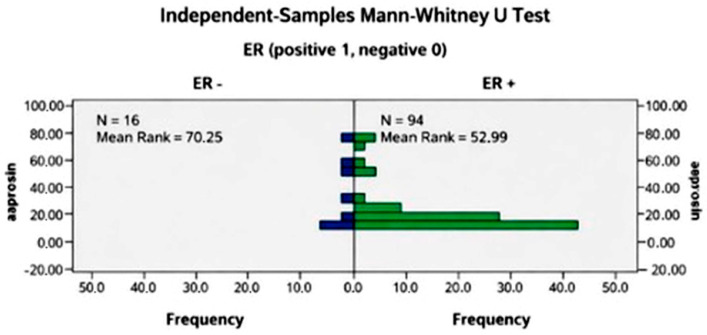
Comparison of serum asprosin levels according to estrogen receptor (ER) status in breast cancer patients. Data are shown as median and interquartile range (IQR). Statistical analysis was performed using the Mann–Whitney U test. Although a numerical difference was observed, the association between serum asprosin levels and estrogen receptor (ER) status was not statistically significant after correction for multiple testing.

**Figure 4:**
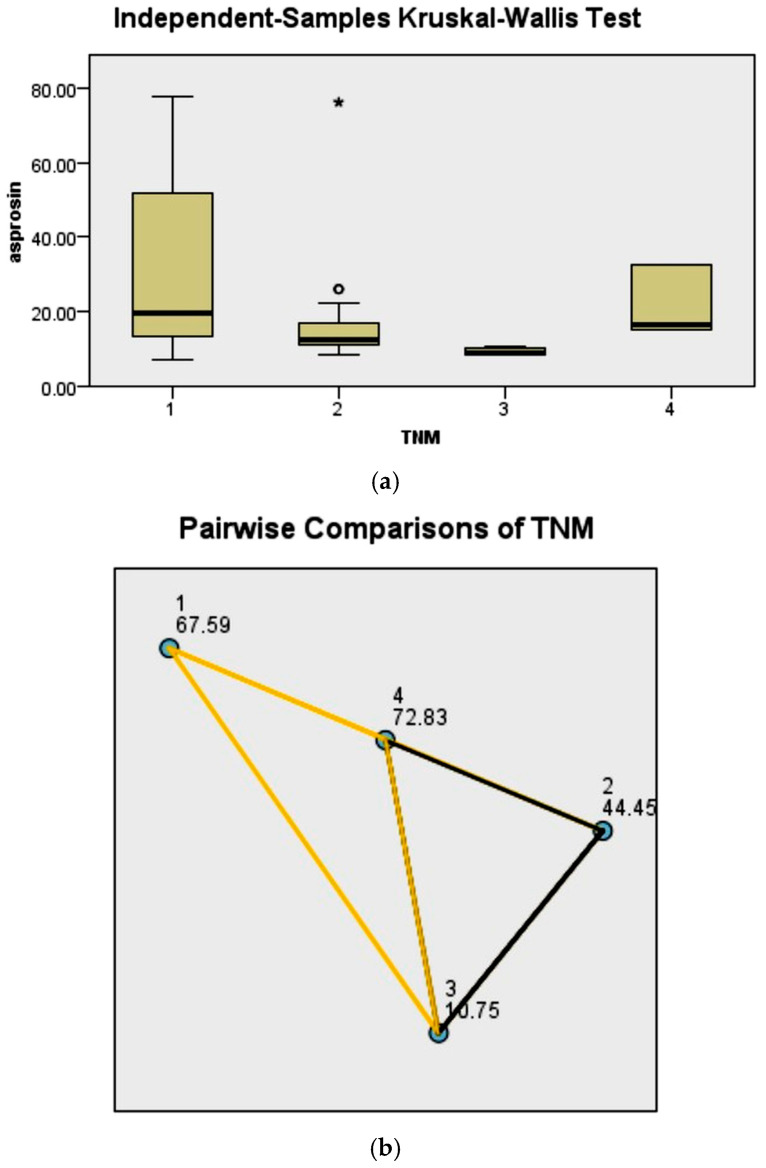
(**a**) Comparison of serum asprosin levels according to axillary lymph node status in breast cancer patients. (**b**) Comparison of serum asprosin levels according to disease stage (Stages 1–4) in breast cancer patients. Data are shown as median and interquartile range (IQR). Statistical analysis was performed using the Kruskal–Wallis test. In subfigure (**a**), the asterisk (*) and dot denote statistically significant pairwise differences between the indicated groups based on post hoc analysis.

**Figure 5:**
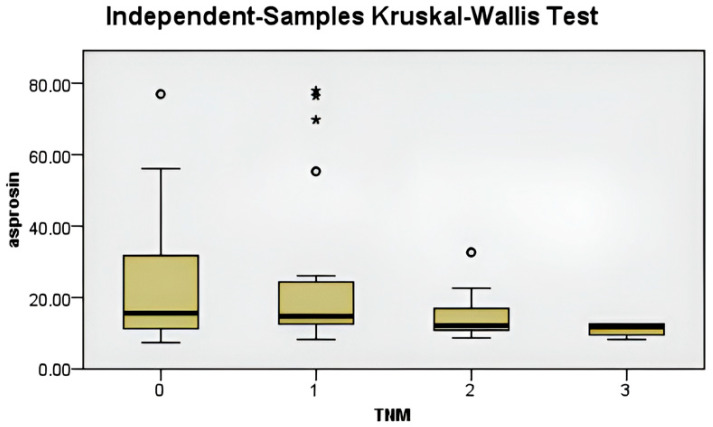
Comparison of serum asprosin levels according to tumor size categories (T1–T4) in breast cancer patients. Data are shown as median and interquartile range (IQR). Statistical analysis was performed using the Kruskal–Wallis test. A statistically significant difference was observed among tumor size groups (*p* < 0.001). The asterisk (*) and dot indicate statistically significant pairwise differences between the specified groups based on post hoc analysis.

**Figure 6:**
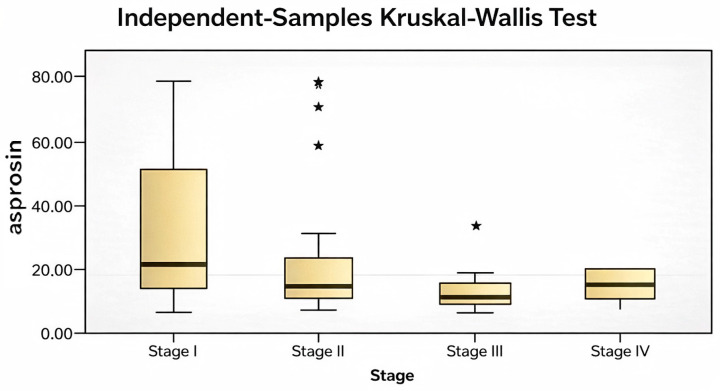
Comparison of serum asprosin levels according to progesterone receptor (PR) status in breast cancer patients. Data are shown as median and interquartile range (IQR). Statistical analysis was performed using the Mann–Whitney U test. The asterisk (*) indicates a statistically significant difference between the compared groups.

Subgroup differences contributing to these findings are illustrated in Figure 3, Figure 4, Figure 5 and Figure 6.

No statistically significant associations were observed between serum asprosin levels and estrogen receptor status or the Ki-67 proliferation index after correction for multiple testing (*p* = 0.083 and *p* = 0.847, respectively).

A statistically significant correlation was found between BMI and serum asprosin levels (Spearman’s rho, *p* = 0.004). A significant correlation was also observed between breast cancer presence and serum asprosin levels (Spearman’s rho, *p* < 0.0001).

For estrogen receptor status, the unadjusted *p*-value did not remain statistically significant after correction for multiple comparisons.

## 4. Discussion

The majority of previously published studies investigating serum asprosin in breast cancer have used retrospective designs and have evaluated clinicopathological associations only to a limited extent. In particular, the study reported by Yur et al. has important methodological limitations regarding causal interpretation due to its retrospective structure and lack of fasting standardization [5]. In contrast, the present study was conducted prospectively with strict fasting standardization and a well-defined healthy control group. Moreover, serum asprosin levels were evaluated not only in relation to tumor presence but also in association with early-stage disease and selected clinicopathological parameters. To our knowledge, this is among the first prospective, fasting-standardized case–control analyses focusing specifically on the relationship between serum asprosin levels and early-stage breast cancer characteristics. In this respect, the present study provides methodologically and clinically complementary data to the existing literature regarding the potential diagnostic role of asprosin in breast cancer.

In the present study, serum asprosin levels were shown to be significantly higher in patients with breast cancer compared with healthy controls. The median serum asprosin level was 14.3 ng/mL in the breast cancer group, whereas this value was 11.0 ng/mL in the control group. The cutoff value of 12.2 ng/mL determined by ROC curve analysis provided a sensitivity of 66.4% and a specificity of 65.5%. These findings indicate that serum asprosin demonstrates statistically significant but moderate accuracy performance in the diagnosis of breast cancer [5,6].

The moderate performance revealed by ROC curve analysis suggests that serum asprosin is not suitable for clinical use as a standalone screening or diagnostic tool. However, it may contribute as a complementary biomarker to existing diagnostic approaches, particularly in early-stage disease. From a screening perspective, prioritizing sensitivity over specificity may be considered in future studies to explore the potential role of serum asprosin as an early detection adjunct. In clinical practice, evaluating serum asprosin levels together with imaging modalities such as mammography may provide additional biological information, especially in the interpretation of suspicious or borderline radiological findings. Similarly, when used in combination with classical tumor markers (e.g., CA 15-3), serum asprosin may increase diagnostic sensitivity by reflecting biological changes occurring in early-stage disease [6,7]. Therefore, the potential clinical role of serum asprosin may lie more in biological characterization and risk stratification rather than primary diagnosis. Nevertheless, further multicenter and comparative studies are required to clearly establish the clinical value of this complementary approach. This comparison was included to position asprosin within the existing biomarker landscape rather than to imply equivalence or direct clinical interchangeability.

In recent years, spectroscopic and optical-based alternative methods for measuring asprosin and similar biomolecules have been described. In particular, Raman spectroscopy, surface-enhanced Raman scattering (SERS), and optical biosensor-based approaches offer high sensitivity potential; however, these techniques are still in the research phase. Their integration into routine clinical practice is limited due to requirements for specialized equipment, high costs, and lack of standardization [8,9]. In contrast, ELISA-based serum measurements provide important advantages in terms of accessibility, cost-effectiveness, and applicability in routine clinical laboratories. Therefore, in the present study, ELISA-based measurement was preferred as a method that is closer to clinical practice and reproducible.

Beyond accuracy performance, one of the notable findings of this study is the association between serum asprosin levels and specific clinicopathological characteristics. Serum asprosin levels were found to be higher in patients with smaller tumor diameter and early-stage disease (Stage I). These observations suggest that serum asprosin may reflect early biological and metabolic adaptations rather than overall tumor burden [4,10]. Taken together, these findings suggest that serum asprosin may serve as a marker of early tumor-related metabolic adaptation rather than a surrogate of tumor burden, thereby providing biologically complementary information beyond previously reported associations.

Although serum asprosin levels tended to be higher in patients without axillary lymph node metastasis, this difference did not reach statistical significance (*p* = 0.050) and therefore should be interpreted with caution and cannot be considered conclusive.

As the disease progresses, structural and metabolic changes in the tumor microenvironment, increased chronic inflammation, and disruption of systemic metabolic balance may contribute to attenuation or exhaustion of the circulating asprosin response. This phenomenon represents one possible explanation for why serum asprosin levels may not increase in parallel with tumor mass despite greater tumor burden in advanced-stage disease [10,11].

The observation that serum asprosin levels tended to be higher in estrogen receptor (ER)-negative tumors is noteworthy. ER-negative breast cancers are generally associated with more aggressive biological features and poorer prognosis [12,13]. However, the association between elevated serum asprosin levels and ER negativity observed in this study suggests a possible relationship that requires cautious interpretation. Given the limited statistical strength of this finding, definitive conclusions regarding this association cannot be drawn based on the present data. Experimental data suggest that asprosin may influence cellular proliferation and apoptosis-related pathways, including ERK1/2 signaling and antioxidant defense mechanisms; however, the relevance and direction of these pathways in breast cancer biology remain to be clarified and should be interpreted as hypothesis-generating rather than confirmatory [14].

A limited association between serum asprosin levels and progesterone receptor (PR), human epidermal growth factor receptor 2 (HER2), and the Ki-67 proliferation index suggests that serum asprosin is not a marker that directly reflects tumor aggressiveness or molecular subtypes. This finding supports the hypothesis that asprosin may be more closely related to early biological and metabolic changes rather than proliferative activity alone [10,11].

Although variability in serum asprosin levels was observed among molecular characteristics, no separate subgroup analysis was performed for triple-negative breast cancer (TNBC) in the present study. Therefore, potential associations between serum asprosin levels and TNBC cannot be evaluated based on the current data, and future studies including specific subgroup analyses are required to address this issue.

Although the number of studies investigating the relationship between asprosin and breast cancer is limited, the present findings are generally consistent with previous reports. A study conducted in Türkiye in 2024 reported higher serum asprosin levels in patients with early-stage breast cancer compared with those with advanced-stage disease [5,15].

In this context, serum asprosin may provide additional information regarding tumor biology and disease stage, distinct from classical tumor markers. In the present study, cancer antigen 15-3 (CA 15-3) was mentioned solely to provide contextual comparison with established biomarkers reported in the literature, and no direct association analysis was performed between serum asprosin and CA 15-3 [6,7].

## 5. Strengths and Limitations of the Study

This study has several important strengths. First, the demonstration of a clear and statistically significant difference in serum asprosin levels between patients with breast cancer and the control group supports the robustness of the main findings. Second, the accuracy performance of serum asprosin was objectively evaluated using ROC curve analysis, and sensitivity and specificity estimates were reported together with the optimal cutoff value. Third, associations between serum asprosin levels and a wide range of clinicopathological parameters, including tumor size, disease stage, lymph node status, and selected molecular characteristics, were comprehensively examined. Finally, to our knowledge, this study is among the pioneering investigations evaluating the relationship between asprosin, a recently identified hormone, and breast cancer using a prospective design in a relatively large and well-defined patient cohort.

The study was conducted at a single center, and all laboratory infrastructure, equipment standardization, and preanalytical and analytical processes were consistently applied within the same institution. While this approach enhances the internal consistency of the measurements, it represents a natural limitation with respect to the generalizability of the results to different laboratory settings.

Despite these strengths, the study also has certain limitations. Although the sample size is comparable to previous studies in this field, it remains relatively limited and may restrict the generalizability of the findings. Due to the limited sample size, multivariate analysis could not be performed; therefore, serum asprosin cannot be considered an independent predictor of breast cancer in the present study. Although patients with diabetes mellitus were excluded, insulin resistance and other metabolic parameters were not systematically assessed and may represent residual confounding factors influencing serum asprosin levels. In addition, the analyses were limited to circulating serum asprosin levels, and tissue-level asprosin expression was not evaluated, precluding direct comparison between systemic and local tumor expression. Furthermore, serum asprosin measurements were obtained at a single preoperative time point, which did not allow evaluation of dynamic changes during disease progression or treatment. The biological source of elevated circulating asprosin levels also remains speculative, as the present study does not allow differentiation between tumor-derived secretion and systemic metabolic responses. The absence of longitudinal follow-up data further precluded assessment of the prognostic value of serum asprosin or its potential role in predicting treatment response or survival outcomes. In addition, although height and weight were recorded for BMI calculation, separate descriptive summaries for these variables were not preserved in the statistical export files; therefore, BMI was used as the primary anthropometric reporting variable.

For these reasons, multicenter, prospective studies incorporating multivariate analyses are required to validate our findings in larger populations and across different laboratory settings. The present study provides foundational, hypothesis-generating data for more comprehensive future research in this field.

## 6. Conclusions

The findings of this study demonstrate that serum asprosin levels are significantly increased in patients with breast cancer compared with healthy controls. While these results suggest that asprosin may represent a potential serum biomarker in breast cancer, validation through multicenter studies with larger sample sizes and multivariate analyses is required before clinical implementation.

Beyond its potential clinical relevance, the associations observed between elevated serum asprosin levels and early-stage disease, smaller tumor size, and concomitant ductal carcinoma in situ suggest that asprosin may reflect underlying tumor biological and metabolic characteristics rather than overall tumor burden. From a clinical perspective, evaluation of asprosin as a non-invasive serum biomarker may offer potential advantages in terms of patient comfort and feasibility. Nevertheless, further prospective studies incorporating tissue-level analyses and long-term follow-up are needed to clarify the clinical utility of asprosin in the diagnosis, monitoring, and risk stratification of breast cancer.

## Figures and Tables

**Table 1:** Clinicopathological characteristics of patients with breast cancer, including histological subtypes (WHO classification).

Characteristics	n	%
Histological type		
Ductal carcinoma (NST)	103	93.6
Invasive lobular carcinoma (ILC)	7	6.4
Tumor grade		
Grade I	10	14.3
Grade II	49	70.0
Grade III	11	15.7
ER status		
ER positive	94	85.5
ER negative	16	14.5
PR status		
PR positive	88	80.0
PR negative	22	20.0
HER2 status		
HER2 positive	33	30.0
HER2 negative	77	70.0
Ki-67 index		
<14%	38	34.5
≥14%	72	65.5
DCIS component		
Present	24	21.8
Absent	86	78.2
Type of surgery		
BCS + SLNB	66	60.0
BCS + ALND	1	0.9
Mastectomy + SLNB	23	20.9
Modified radical mastectomy	20	18.2

**Table 2:** Demographic and menopausal comparison between breast cancer patients and the control group.

Variable	Breast Cancer Patients (*n* = 110)	Control Group (*n* = 110)	*p* Value
Age (years), median (IQR)	51 (45–62)	49 (45–53)	0.014
Body mass index (kg/m^2^), median (IQR)	25.8 (23.7–30.0)	25.75 (23.7–29.2)	0.909
Serum asprosin (ng/mL), median (IQR)	14.3 (11.44–22.6)	11.0 (9.16–13.56)	<0.001
Menopausal status			0.058
Premenopausal, n (%)	52 (44.1)	66 (55.9)	
Postmenopausal, n (%)	58 (56.9)	44 (43.1)	

Note: Continuous variables are presented as median (IQR) and were compared using the Mann–Whitney U test. Categorical variables were compared using the chi-square test. BMI was used as the primary anthropometric variable in the comparative analysis. Height and weight measurements were recorded for BMI calculation; however, their separate descriptive summaries were not retained in the exported statistical dataset.

**Table 3:** Comparison of groups according to serum asprosin cut-off value (12.2 ng/mL).

Serum Asprosin Level (ng/mL)	Breast Cancer Group (*n* = 110)	Control Group (*n* = 110)	
≤12.2, *n* (%)	37 (33.9)	72 (66.1)	*p*-value < 0.001
>12.2, *n* (%)	73 (66.1)	38 (34.2)	

Note: Comparisons between groups were performed using the Pearson chi-square test.

**Table 4:** Pairwise comparison of serum asprosin levels according to HER2 status in breast cancer patients.

Comparison	*n* (HER2+/HER2−)	Statistical Test (Pre-Bonferroni)	Unadjusted *p*-Value	Bonferroni-Adjusted *p*-Value
HER2 (+) vs. HER2 (−)	16/35	Mann–Whitney U test	0.021	0.042

Note: Pairwise comparisons were performed using the Mann–Whitney U test prior to Bonferroni correction. Unadjusted and Bonferroni-adjusted *p*-values are reported to control for multiple testing. A *p*-value < 0.05 was considered statistically significant.

**Table 5:** Comparison of serum asprosin levels according to clinicopathological parameters in breast cancer patients.

Clinicopathological Parameter	Category (*n*)	*p*-Value
Tumor size (TNM)	T1 (*n* = 51); T2 (*n* = 49); T3 (*n* = 4); T4 (*n* = 6)	<0.001
Lymph node status (TNM)	N0 (*n* = 46); N1 (*n* = 39); N2 (*n* = 21); N3 (*n* = 4)	0.050
Stage (TNM)	Stage I (*n* = 31); Stage II (*n* = 50); Stage III (*n* = 25); Stage IV (*n* = 4)	0.020
Tumor grade	Grade I (*n* = 6); Grade II (*n* = 43); Grade III (*n* = 2)	0.193
Estrogen receptor (ER)	Negative (*n* = 10); Positive (*n* = 41)	0.083
Progesterone receptor (PR)	Negative (*n* = 13); Positive (*n* = 38)	0.034
HER2 status	Negative (*n* = 35); Positive (*n* = 16)	0.033
Ki-67 index	<14% (*n* = 20); ≥14% (*n* = 31)	0.847

Note: Comparisons between groups were performed using the Kruskal–Wallis test for variables with more than two categories and the Mann–Whitney U test for two-category variables. Data are presented as median and interquartile range (IQR). A *p*-value < 0.05 was considered statistically significant.

## Data Availability

The data are included in the article. Further inquiries can be directed to the corresponding author.

## Abbreviations

The following abbreviations are used in this manuscript:

**ALND**	Axillary Lymph Node Dissection
**BCS**	Breast-Conserving Surgery
**BMI**	Body Mass Index
**CA 15-3**	Cancer Antigen 15-3
**DCIS**	Ductal Carcinoma In Situ
**ELISA**	Enzyme-Linked Immunosorbent Assay
**ER**	Estrogen Receptor
**HER2**	Human Epidermal Growth Factor Receptor 2
**PR**	Progesterone Receptor
**ROC**	Receiver Operating Characteristic
**SLNB**	Sentinel Lymph Node Biopsy
**SPSS**	Statistical Package for the Social Sciences
**TNM**	Tumor–Node–Metastasis
